# Impact of distance-learning training on substance use screening and brief intervention among health professionals and on their beliefs and attitudes toward drug use

**DOI:** 10.1186/1940-0640-7-S1-A87

**Published:** 2012-10-09

**Authors:** Ana Paula L Carneiro, Denise De Micheli, Monica Maino, Jose Carlos Fernandes Galduroz, Yone Moura, Paulina CAV Duarte, Maria Lucia O Souza-Formigoni

**Affiliations:** 1 Department of Biological Sciences, Federal University of São Paulo, São Paulo, Brazil; 2 Department of Psychobiology, Federal University of São Paulo, São Paulo, Brazil; 3 Center of Information on Psychotropic Drugs, Federal University of São Paulo, São Paulo, Brazil; 4 Brazilian National Secretariat for Policies on Drugs, Rio de Janeiro, Brazil

## 

Screening for alcohol and other drug (AOD) use followed by brief intervention (SBI) represents a useful tool for health professionals, since most people who are in the early stages of substance-related problems receive no guidance before developing significant consequences. In order to disseminate the techniques of SBI among Brazilian health professionals, the National Secretary on Drug Policy (SENAD), in partnership with the Drug Dependence Unit of UNIFESP, developed the distance learning program SUPERA. The aim of this study was to assess whether health professionals who participated in SUPERA changed their beliefs and behaviors related to AOD after completing the training. Health professionals from the Brazilian public health network who successfully completed the course (N = 1062) participated in the study. They answered a questionnaire on their beliefs and attitudes regarding AOD use before and after the course. After completing it, 91% of participants reported feeling more able to use SBI techniques than before, and 60% (compared with 37% pre-training) reported believing that demonstrating concern for patients’ AOD use could help reduce their consumption. Seventy-three percent (versus 50% pre-training) believed in the importance of BI to reduce AOD use, and 60% (versus 30%) reported believing in patients’ capacity to reduce AOD use. Most of the participants (66% after training versus 28% before) considered themselves to have an adequate level of knowledge about AOD use, and 83% (versus 22%) reported high confidence in their ability to detect AOD use. These data indicate positive changes in health professionals’ knowledge and attitudes regarding AOD use after the course, suggesting that distance learning is adequate to train health professionals in SBI.

